# FEM Simulation of the Riveting Process and Structural Analysis of Low-Carbon Steel Tubular Rivets Fracture

**DOI:** 10.3390/ma15010374

**Published:** 2022-01-05

**Authors:** Jaroslaw Jan Jasinski, Michal Tagowski

**Affiliations:** 1 Materials Research Laboratory, National Centre for Nuclear Research, 05-400 Otwock, Poland; jaroslaw.jasinski@ncbj.gov.pl; 2 Faculty of Mechanical Engineering and Computer Science, Czestochowa University of Technology CUT, 42-200 Czestochowa, Poland

**Keywords:** metal forming, riveting, low-carbon steel, FEM simulation, microstructure, tertiary cementite Fe_3_C_III_

## Abstract

Riveted joints are a common way to connect elements and subassemblies in the automotive industry. In the assembly process, tubular rivets are loaded axially with ca. 3 kN forces, and these loads can cause cracks and delamination in the rivet material. Such effects at the quality control stage disqualify the product in further assembly process. The article presents an analysis of the fracture mechanism of E215 low-carbon steel tubular rivets used to join modules of driver and passenger safety systems (airbags) in vehicles. Finite element method (FEM) simulation and material testing were used to verify the stresses and analysis of the rivet fracture. Numerical tests determined the state of stress during rivet forming using the FEM-EA method based on the explicit integration of central differences. Light microscopy (LM), scanning electron microscopy (SEM) and chemical composition analysis (SEM-EDS) were performed to investigate the microstructure of the rivet material and to analyze the cracks. Results showed that the cause of rivet cracking is the accumulation and exceeding of critical tensile stresses in the rivet flange during the tube processing and the final riveting (forming) process. Moreover, it was discovered that rivet fracture is largely caused by structural defects (tertiary cementite Fe,Mn_3_C_III_ along the boundaries of prior austenite grains) in the material resulting from the incorrectly selected parameters of the final heat treatment of the prefabricate (tube) from which the rivet was produced. The FEM simulation of the riveting and structural characterization results correlated well, so the rivet forming process and fracture mechanism could be fully investigated.

## 1. Introduction

The use of rivets in the assembly process is widespread. The riveting process is often used to join components in many industries, i.e., automotive and aerospace. Such connection is usually the most sensitive point in the mounting structure. It is a fastening technique where a joint is created between the materials with the application of a die/stamp. This process joins different materials (i.e., metal-polymer) through the formation of mechanical interlock [1,2,3,4]. The essential properties of the rivets are enough hardness and ductility to realize an accurate forming process and avoid cracking [5,6]. Nevertheless, there are few requirements to complete when producing and forming tubular rivets, such as proper microstructure of the steel, controlled machining and cutting of the rivets, and estimation of the stamping force during riveting [7,8]. However, for investigation of the plastic behavior of the clip, it is beneficial to simulate the stress distribution to estimate the risk of rivet fracture and to describe the stresses linked to each production process stage and critical stresses occurring during riveting. Blanchot and Daide indicate that it is crucial to consider initial riveting phase. During the initial phase, rivet due to high stress value, is exposed to microcracks and thereafter to propagation cracking [9,10]. Finite element method (FEM) analysis allows verifying process parameters and tool geometry, which may significantly influence the creation of initial microcracks during riveting. Bedair and Eastaugh state that 3D finite element (FE) models may better represent the stress distribution around rivet holes [11,12]. However, extensive modelling efforts and computer time are normally required, which may not be practical for analyzing structures with a large number of rivets. Nevertheless, in cases of assembling components related to user safety, it is crucial to analyse the FE model as close as possible to real conditions. During analysis the average computational time was ~52 h [13,14,15,16].

**Figure 1:**
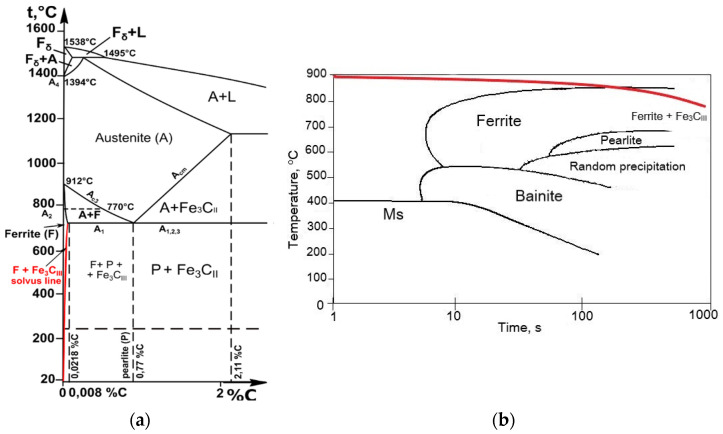
Transformations in equilibrium state and during cooling of low carbon steels, (**a**) Fe-Fe_3_C phase diagram [22], (**b**) CCT diagram presenting slow-cooling after austenitizing [23], (red curves symbolize the precipitation regions of Fe_3_C_III_ phase).

Moreover, a fundamental understanding of the rivet’s materials microstructure evolution from solidification to the final product is also critical. Low-carbon unalloyed steels microstructure depends on the chemical composition, and of course on the solidification conditions and final heat treatment process realized [17,18,19]. Properties such as hardness and tensile strength can be increased through correct heat treatment (annealing and cooling). However, certain process errors can occur during the heat treatment of low-carbon unalloyed steel and thus reduce its mechanical properties. One such mistake is the inadequate choice of the cooling rate after annealing and the extension of the austenitizing time leading to austenite grain growth [20,21]. For such heat treatment analysis of the material, the continuous cooling transformation (CCT) diagram and Fe-Fe_3_C diagram are useful to study transitions in iron-carbon alloys during cooling and examine microstructures in an equilibrium state (Figure 1).

According to the phase diagram, alloys containing up to 0.1% C are two-phase alloys with a ferrite and pearlite structure. The solvus line in Figure 1a (red curve) shows that the solubility of carbon in ferrite decreases with decreasing temperature, i.e., from 0.0218% C at 727 °C to <0.008% C at 20 °C. Ferrite, during slow cooling from 727 °C (eutectoid temperature) to room temperature 20 °C, as shown in Figure 1b, leads to the precipitation of cementite called tertiary cementite (Fe_3_C_III_) along the boundaries of previous austenite grains. Such structure causes cleavage microcracks in steels and is usually considered the factor responsible for embrittlement in low carbon steels [24,25]. Tertiary cementite separates from ferrite during slow cooling below A_r1_ temperature due to the variable solubility of carbon in Fe_α_ along the solvus line. Therefore, when the carbon content of slowly cooled low carbon steels increases, the proportion of cementite increases and the share of ferrite decreases. These changes in the microstructure of the steel cause significant changes in its mechanical properties. The tensile strength of ferrite in low carbon steels after the cold drawing is about 420 MPa and the hardness HB approximately 110, while cementite has a hardness of HV ≈ 700 and almost no tensile properties [26]. This type of equilibrium structure significantly affects the low mechanical properties of the steel, as well as its susceptibility to cracking. Ferrite constantly forms at austenite grain boundaries during slow cooling. The atoms at the grain boundaries are not in lattice sites and are in a high-energy state, so these are the sites of the beginning of ferrite formation, but this is accompanied by the simultaneous release of tertiary cementite at these sites [27,28]. Therefore, the leading cause of embrittlement in low carbon steels is related mainly to cooling from the austenitizing temperature. The cooling rate is a decisive factor of structural changes and changes related to the mechanical properties of low carbon steel. It is worth noting that during faster cooling (rapid air cooling), embrittlement of the material does not occur. On the other hand, in most cases the segregation of Mn and Si elements is considered a factor leading to a decrease in cohesive strength of grain boundaries and fracture of the material [29,30,31]. This paper aims to analyze the fracture of tubular rivets through structural characterization and finite element simulation and describe the fracture mechanism occurring during the riveting process. The research methodology combines an experimental approach with a computational simulation of the riveting process.

## 2. Materials and Methods

The rivet material was E215 low carbon steel with the chemical composition presented in Table 1. The structure of the material and chemical composition of the inclusions and precipitates was examined using a light microscopy LM (Carl Zeiss Axiovert, Jena, Germany) and scanning electron microscopy SEM-EDS (JEOL 6600, Peabody, MA 01960, USA). The microstructural observations were made on sections after grinding from 320 to 2000 grit and polished with diamond suspensions of 9 μm, 3 μm and 1 μm and 3% Nital etched. Structural analysis was realized to determine the distribution of Fe_3_C precipitates and non-metallic inclusions and investigate crack initiators with fracture types. Hardness tests of rivets surface and flange were realized using a microhardness tester (Future-Tech FM-7, Kawasaki, Japan) with a testing force of 0.2452 N (HV0.025).

**Figure 2:**
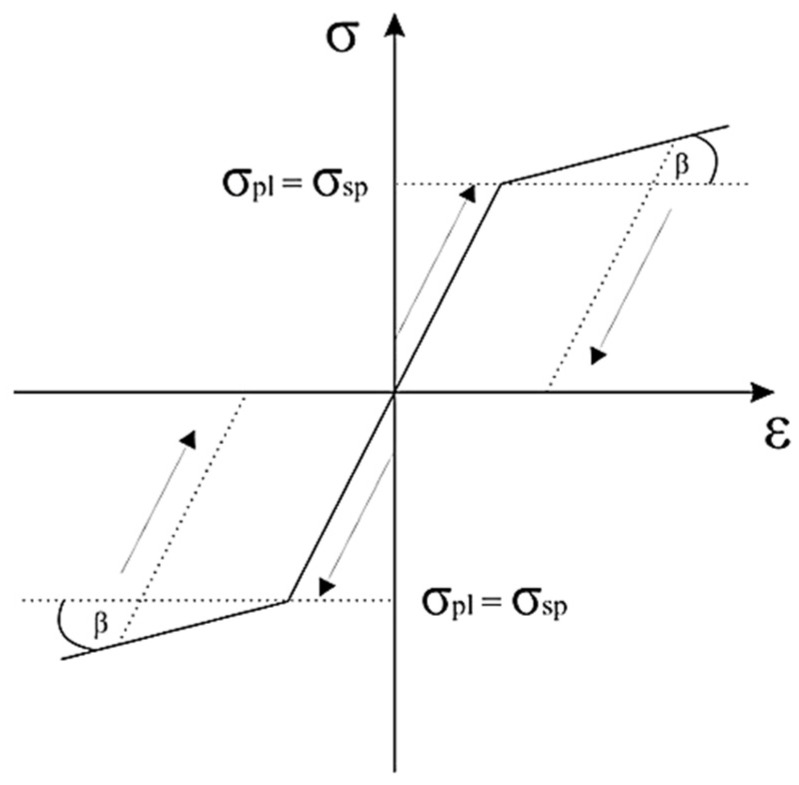
Prandtl-Reuss model curve σ = f(ε).

Computer simulation of the rivet forming process was performed by the FEM method using Abaqus software. The numerical simulation of the entire process was performed to verify the correct selection of the riveting process force and control the tool (die/stamp) geometry, which excludes the possibility of rivet fracture. For this purpose, the method of explicit integration of central differences was used. This procedure is characterized by conditional convergence. This means that the size of the time step ∆t is smaller, the smaller the size of the generated mesh of finite elements. During the calculations, the displacements of nodes (according to the degrees of freedom u^N^) in the analyzed model for the time moment t + ∆t were determined based on the solutions obtained in the previous calculation steps (Figure 2).

The Prandtl material model assumes that the plastic deformations are so limited that elastic deformations constitute a significant part of the total deformations and, therefore, cannot be ignored. After exceeding the yield point, plasticity may be ideal or with linear hardening depending on the material’s behaviour. The yield point for material without hardening is a constant value. Prandtl-Reuss equations present the flow rule for an ideally elastic-plastic material. These equations show the relation between a plastic strain increment and the stress deviator. If the considered strain is significant, the elastic increment is negligible concerning the total pressure, and the Prandtl-Reuss equations become the Levy-Misses equation.

**Figure 3:**
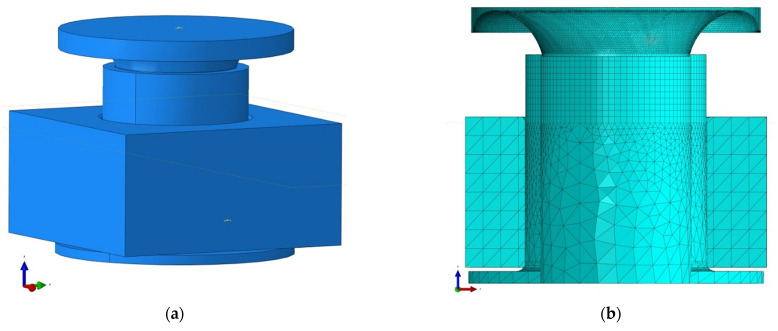
FE model assembly stage (**a**) view of the instance model, (**b**) cross-section with visible finite element meshes.

The process of rivet forming includes three phases: elastic deformation, plastic deformation and finally, springback stage. The issue of such a process was considered as a contact issue. Two definitions of contact were defined: general contact (between the rivet and joined details) and the surface-surface contact (tool-rivet pair). General contact allows defining interactions between several or all regions of the model under consideration, using a single definition. Contact surface-surface enables the definition of the interaction between two deformable surfaces or between a deformable surface and a perfectly rigid surface. Based on the geometry of perfectly rigid details, a mesh of triangular three-node elements (R3D3) was created. The rivet was divided into two partitions, on which a mesh of two types of elements was built. The eight-nodes cubic mesh (C3D8R) compacted in the rivet forming area and the tetrahedral mesh (C3D4) with a greater inter-node distance from the base to the cylindrical part-below the upper edge of the joined details. Tetrahedral and cubic elements are universally used in mesh generator algorithms. Complex shapes can be easily described by means of tetrahedral meshes. Still, since the derivatives of the shape functions are constant with respect to the element’s volume, the results of the derivatives obtained in such element may be wildly inaccurate. Hence, in the case under consideration, the division into upper (significant cubic) and lower (less critical from the point of view of the case under consideration-tetrahedral) partition. The assembly of the instance model and the view of the finite element meshes are shown in Figure 3.

The riveting force and the spatial displacement of the rivet node during the process were also analyzed. Creating the characteristics of force-displacement curves for the rivets is also presented (see Figure 7).

## 3. Results and Discussion

### 3.1. FEM-EA Simulation of the Riveting Process

**Figure 4:**
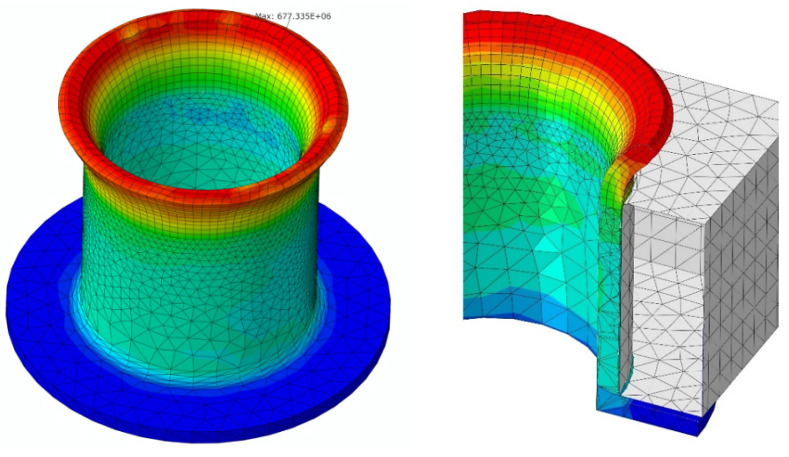

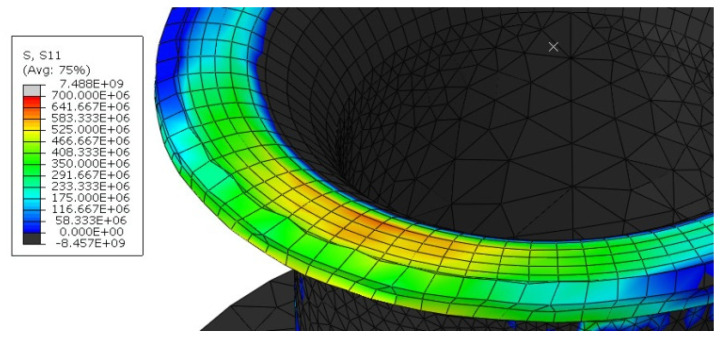
FEM-EA simulation of the initial phase of the riveting process.

As mentioned, the riveting process comprises three phases: the elastic deformation of the rivet, then its plastic deformation and finally the de-stressing of the rivet after the force is removed from the tool. The static axial tensile test provides yield strength and ultimate tensile strength. The values obtained from specimens were: R_m_ ~ 495 MPa, R_e_ ~ 320 MPa. The tool’s geometry caused a dangerous increase in stress c.a. 677 MPa (reduced) in the area of the upper outer edge of the rivet at the start of the process as shown in Figure 4. Component S11 of tensile stress c.a. 580 MPa, provided from analysis, is far beyond R_m_ of E215 steel, which might cause the fracture.

**Figure 5:**
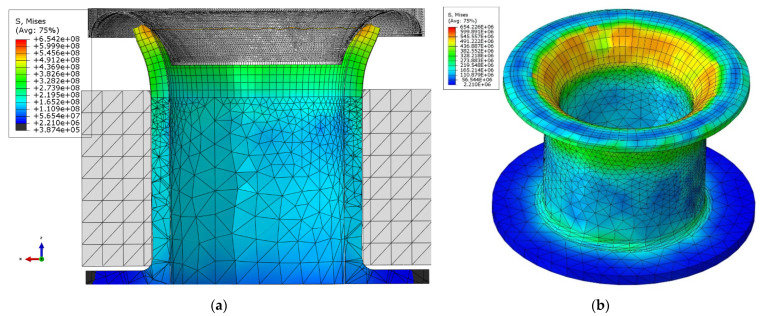
FEM simulation results (**a**) rivet cross-section of the rivet and tool with modified geometry during the riveting process, (**b**) final result after riveting.

After modifying the tool geometry, the results show that the applied process parameters and tool geometry are correctly chosen and should not cause cracks in the rivet’s forming area (upper surface) as shown in Figure 5.

**Figure 6:**
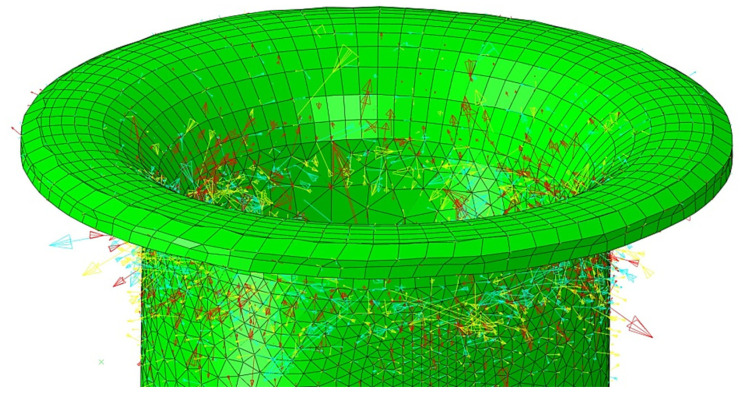
The direction of stress at the end stage of the forming process.

Figure 5 shows the rivet cross-section’s stress distribution and the final rivet after the forming process. However, also important in the evaluation of the riveting process is the stress distribution on the outside of the riveted flange, which was also calculated and presented in Figure 6.

**Figure 7:**
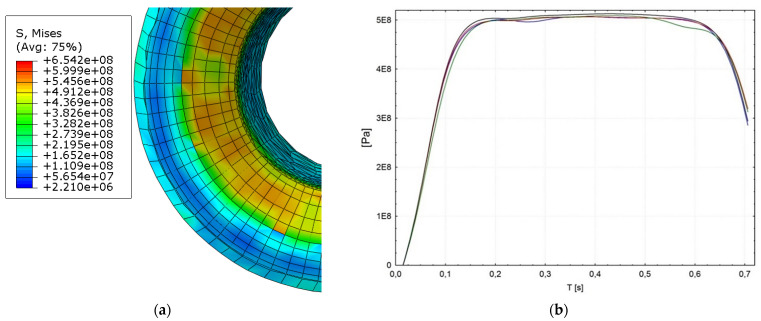
Bending stage of the tubular rivet (**a**) flange of the rivet, (**b**) reduced stress values in the few nodes at the outer, upper edge during the riveting process.

It can be seen that the total stress in the riveted flange was tensile, and the maximum stress was ca. 500 MPa. This stress originated at the final stage of the riveting process, which occurred at the bending stage of the joint flange. This stress originated in the final phase of the riveting process, which occurred during the bending phase of the joint flange. Figure 7 shows that the magnitude of the residual stress in the rivet flange was higher in the area compared to the stress outside the rivet flange.

**Figure 8:**
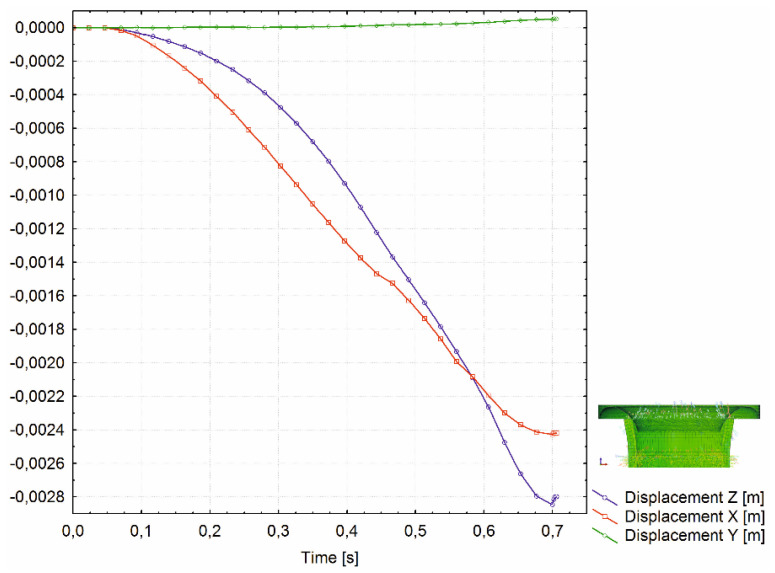
Spatial displacement of node on OD of rivet during process XZ plane.

The tensile stresses significantly increased from the beginning of the flange bend to the final surface of the flange. In contrast, compressive residual stresses rose slowly in the face of the flange to the final surface of the flange. Also, the spatial displacement of the nodes on the outer diameter (OD) of the analyzed rivet part shows smooth transition during each of three process phases (during elastic and plastic deformation and tool retraction). Figure 8 shows the displacement of the node on the OD on XZ plane.

Results showed that the total stress in the inner wall of the rivet shell was tensile and symmetrical about the centre of the rivet. The magnitude of this stress was 420 ± 27 MPa. It is reasonable to assume that this stress originated in the initial phase of rivet shaping. It can also be seen in Figure 7 and Figure 8 that the tensile stress gradually increased towards the rivet head, which finally led to a fracture. 

### 3.2. Structural Analysis of Rivets Fracture

**Figure 9:**
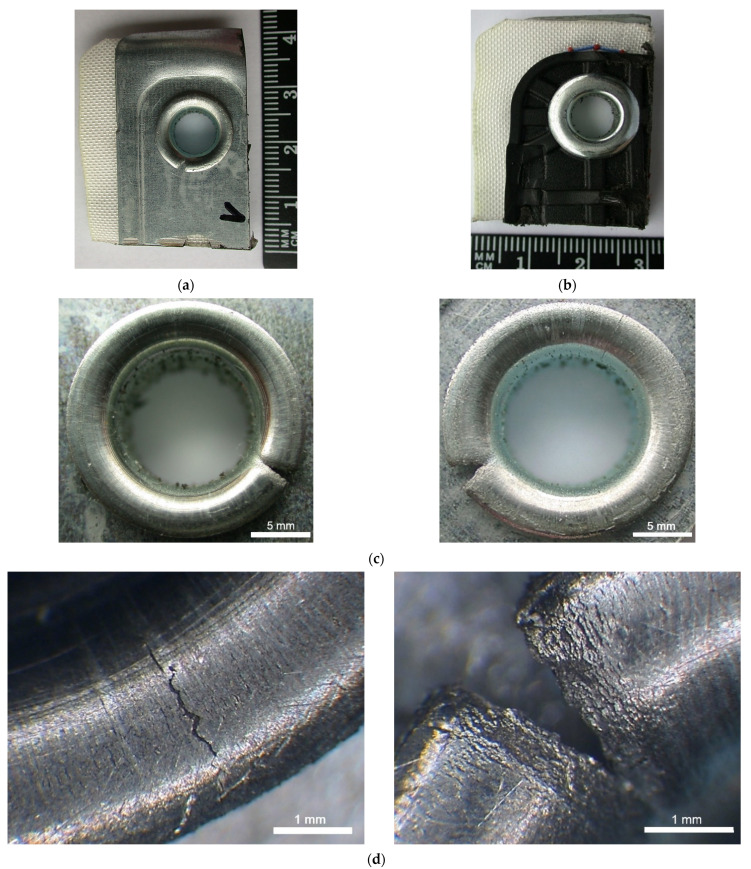
Macroscale image of rivets after rupture (**a**) cross-section of airbag module with visibly broken rivet—top side of module), (**b**) cross-section of airbag module—bottom side of module, (**c**) image of the broken rivet, (**d**) fracture surface of broken rivet after forming process.

In general, the study aimed to analyse the causes of rivet fracture during the manufacturing process and assembly of vehicles airbag module components. The results of FEM simulations confirmed that the riveting process force and parameters were chosen correctly. However, strong local plastic deformations accompanying the riveting process result in tensile stresses, leading to rivet cracks and disqualifying the airbag module component. Therefore, a microstructural analysis of the rivet flange areas was carried out. Macroscopic observations showed that compressive stresses gradually increase in the area under the flange, while critical tensile stresses occur at the rivet flange surface, ultimately leading to rivet fracture. Macroscopic observations of the rivets after the forming process show that cracks of varying lengths appear on the surface of the rivet flange after expansion, occurring transversely to the forming direction of the rivet material. The observed defects are a consequence of the accumulation of stresses and structural defects in the rivet material. Chipping of the rivet material, wedge cracks and permanent mechanical deformation effects were also observed in the flange, mainly in the tensile stress area. Cracks were observed in the elongated rivet flange, as shown in Figure 9, and such effect is related to the almost flat force gradient in the flange during the rivet forming stage.

**Figure 10:**
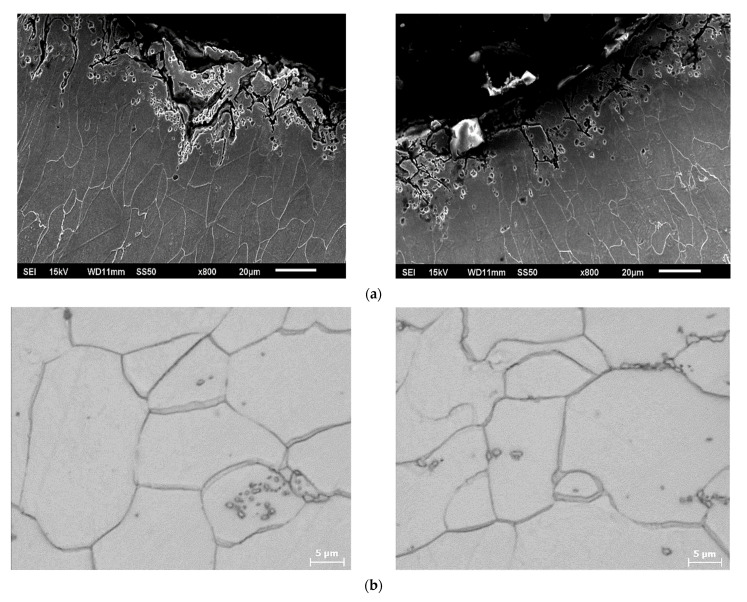

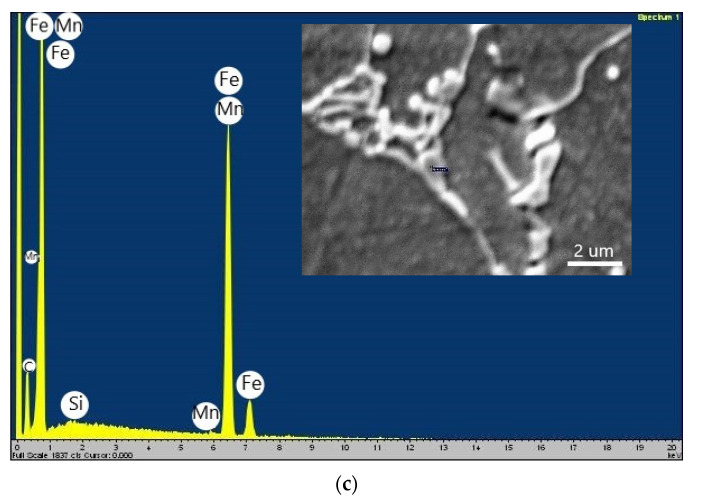
Microstructure of the rivet after fracture during the forming process (**a**) rivet flange surface (SEM) (**b**) visible Fe_3_C_III_ tertiary cementite through grain boundaries (LM), (**c**) chemical analysis of (Fe,Mn)_3_C_III_ tertiary cementite (SEM-EDS).

The microstructure observation of the rivet matrix material indicates that it is made of low carbon steel used for precision seamless tubes. The structure of the core material of the rivets is ferrite with carbides separations as spheroidal carbides, bone-like carbides and tertiary cementite (Fe_3_C_III_) at the grain boundaries, as shown in Figure 10. The ferrite grains showed considerable variation in size and effect of recrystallization twins in the ferrite matrix, confirming the annealing process in which the cooling rate was too slow. Such microstructural heterogeneity also favors crack initiation. Further observation by scanning electron microscope and semi-quantitative analysis of the carbides and inclusions by SEM-EDS showed that the composition of the precipitates is mainly Fe_3_C_III_ and Fe,Mn_3_C_III_ compounds. The carbide phase separated during the heat treatment process, which is a brittle phase, and in this case determines the reduction of the rivet material strength and cracking during the riveting process.

**Figure 11:**
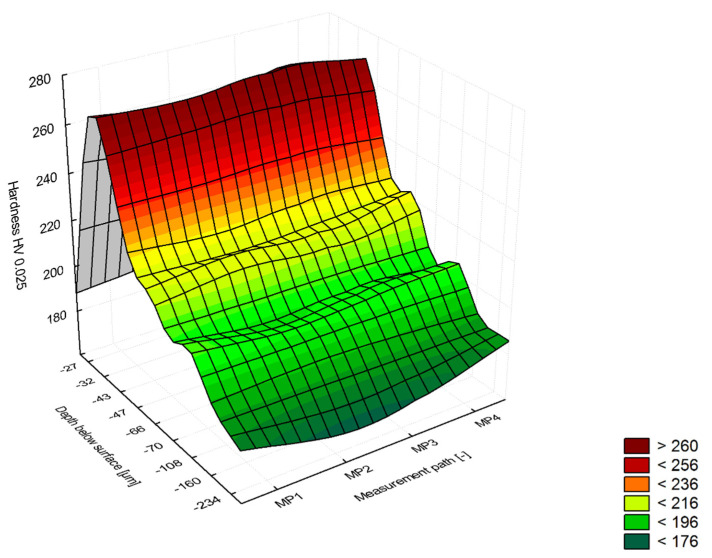
Microhardness HV0.025 measurements results of the E215 low-carbon steel rivets after riveting process.

Observation of the E215 steel microstructure, analysis of the shape and distribution of the precipitates also indicates that the material was annealed. However, when there is a long cooling process, the tertiary cementite will be precipitated both in the surface layer and in the matrix. This effect indicates that the annealing treatment has not been properly carried out. The hardness tests also supplemented the analysis of the microstructure. The hardness in the core of the rivet is from 130 to 170 HV0.025, while the maximum hardness in the strengthened zone (flange surface) was from 250 to 270 HV0.025 at a distance of approx. 50 μm from the surface in the area of maximum stress after riveting (Figure 11). 

**Figure 12:**
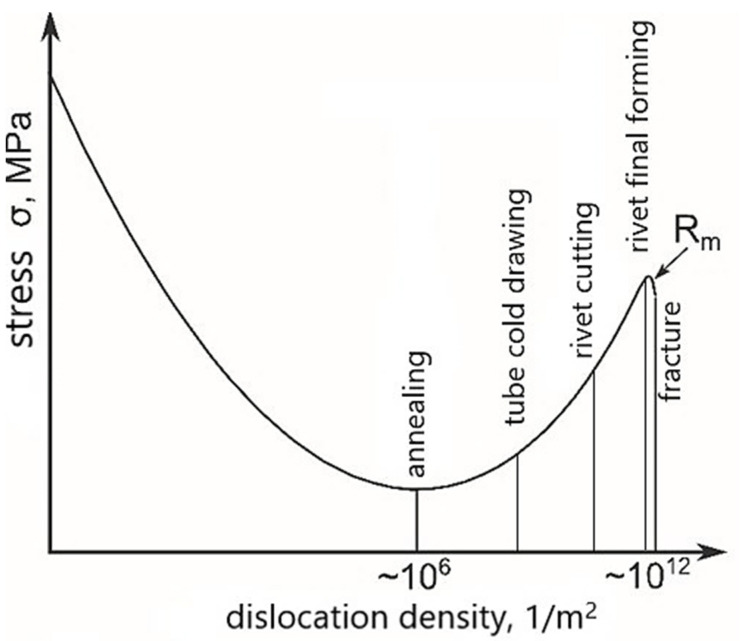
Effect of surface layer defects on the strength properties of the rivet material after various stages of the production process and final riveting.

The grains deformation occurring in the surface layer of the rivets results from the mentioned stresses consequently by the accumulation of defects in each stage of the production process, which is schematically presented in Figure 12. The rivet breaks when defects accumulate in the mechanically deformed surface layer and the critical tensile strength (R_m_) is exceeded. Successive technological processes influence the accumulation of defects: cold drawing of the pipe, then cutting and shaping of the rivet head, and finally forming during assembly.

**Figure 13:**
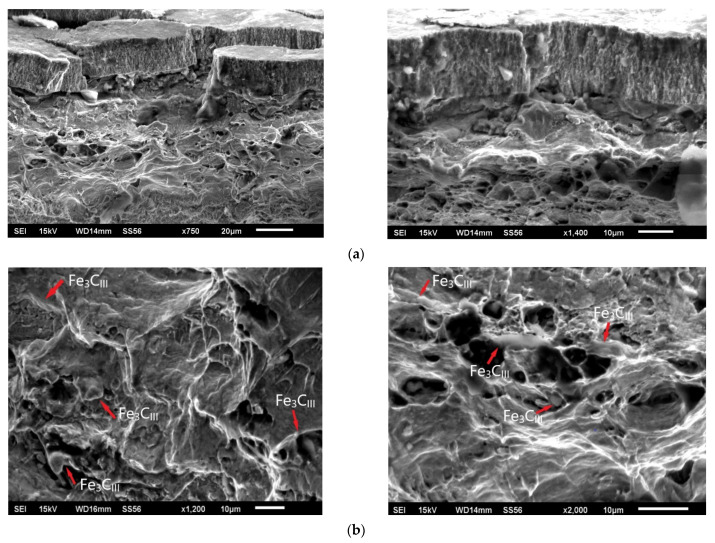
SEM image of rivet fracture after forming process, (**a**) surface of rivet flange (**b**) fracture structure with visible tertiary cementite (Fe_3_C_III_) phase (red markers).

In tensile-compression cracking, the loads generated during forming of low-carbon steel in the longitudinal direction (rivets forming process) promote the nucleation of cracks along grain boundaries of ferrite, which in this case are initiated by the net of tertiary cementite at grain boundaries [32,33,34]. Nucleation cracks at Fe_3_C_III_ have been shown to play a decisive role here. The increased proportion of such precipitates in the delivery state of the E215 material induces local stresses and microcracks at the ferrite-tertiary cementite interface. In the rivet forming process, the increased tensile forces induce microstructure rupture and rivet destruction. SEM method was used for the fractographic analysis of the rivet fracture. Both surface and matrix areas mainly exhibited intergranular fracture through grain boundaries and significant grain boundary decohesion. The rivet fracture analysis also showed permanent mechanical deformation effects, mainly in the tensile stress zones. The rivet breakthrough is ductile type, but there are also noticeable primary cracks that spread on the rivet’s surface, revealed after mechanical trimming the pipe to the nominal size. Such effect may also indicate significant wear of the cutting tool when cutting the rivet to the required size (Figure 13).

Analysis of the rivet fracture mechanism indicates that the breaking of bonds follows the separation of the material during rivet formation in areas beyond the strength limit, which in the subsequent expansion stage (forming) reveals pronounced cracks spreading from the rivet flange surface in depth. The cracks beneath the delamination are broader and more extended, indicating the susceptibility of this zone to fracture, along grain boundaries favored by tertiary cementite precipitates. In contrast, cracks above the delamination area propagate towards the rivet surface for a distance of approximately 8÷15 μm. The obtained microstructure results of the surface layer and core are well correlated with the obtained FEM simulation results.

## 4. Conclusions

The paper characterises the riveting process using FEM simulation and carries out structural studies on E215 low-carbon steel tubular rivets fracture. The obtained stress distributions during the riveting process simulation show the tensile stresses in the rivet flange and compressive stresses in the lower part of the rivet head. The performed simulations suggest that as a result of the shape of the tool (die/stamp) the value of the forming force can be varied to limit the occurrence of critical tensile stresses. Performing FEM simulations provides essential insights into the steel forming process that can be used to optimize riveting process parameters. The FEM results of riveting process showed a correlation with changes in material microstructure after heat treatment of E215 low carbon steel, providing a better understanding of the fracture analysis. One of the causes of rivet cracks is mechanical damage and the accumulation of defects in the surface layer and structure of the rivets after subsequent technological processes, i.e., cold drawing of the pipe, cutting to nominal size and forming of the rivets in the production and assembly process. The flaws in the rivet are also areas of tensile stress concentration which lead the rivet material to crack as a result of the loads applied during forming. Apart from stress-related defects, the primary cause of cracking includes the material’s structural inhomogeneity, as tertiary cementite separations in the boundaries of ferrite grains, and the presence of non-metallic inclusions (sulphides, oxysulphides). The tertiary cementite separations result from an incorrectly performed heat treatment process, consisting of a too slow cooling rate after annealing and entering the Fe_3_C_III_ region on the solvus line according to the presented CCT and Fe-Fe_3_C diagrams. In general, when the critical temperature (T_Ar1_) is reached, the subsequent cooling rate must be controlled to avoid the formation of Fe_3_C_III_ (low cooling rates below T_Ar1_ = 727 °C must be avoided). To limit the effect of accumulation of defects in rivet machining processes, it is suggested to make them from properly annealed material (preferably in a protective atmosphere and faster cooled). Such a delivery condition of pipes will not completely eliminate the effect of machining defects, but definitely will eliminate the brittle tertiary cementite phase and improve the strength of the material. After the production process of the rivet (tube drawing, cutting), stress relief annealing is also recommended so that the level of material plasticity is sufficient for forming the rivet in the assembly process. The realized FEM simulation of the riveting process and the rivet fracture structural analysis will ultimately allow for the improvement of the rivet processing, elimination of structural defects and reduction of rejects in the assembly during production process.

## Figures and Tables

**Table 1:** Chemical composition of E215 low-carbon steel used for rivets manufacturing.

Steel Grade	Element, % Mass						
E215	C	Si	Mn	P	S	Al	Fe
	0.10	0.05	0.70	0.020	0.025	0.025	balance

## Data Availability

The data presented in this study are available on request from the corresponding author. The data are not publicly available due to possibility for use in further research.
